# Relationship between collateral flow and exercise performance in Fontan patients: an exercise CMR study

**DOI:** 10.1186/1532-429X-17-S1-O93

**Published:** 2015-02-03

**Authors:** Kevin K Whitehead, Matthew A Harris, Stephen M Paridon, Elaine Tang, Zhenglun (Alan) Wei, Ajit P Yoganathan, Mark A Fogel

**Affiliations:** 1 Children's Hospital of Philadelphia, Sewell, PA, USA; 2 Engineering, Georgia Institute of Technology, Atlanta, GA, USA

## Background

We have recently demonstrated that Fontan geometry and power loss have important relationships with exercise performance. Systemic to pulmonary arterial collateral flow (CollF) has been shown to be an important consideration in single ventricle physiology. However, the effect of exercise on CollF and the relationship between CollF and exercise performance have yet to be studied systematically. We utilized an exercise CMR protocol to investigate the relationship between exercise and CollF.

## Methods

37 Fontan patients prospectively underwent a metabolic exercise protocol followed by a resting and exercise CMR. Total ascending aortic (Ao), superior vena caval (SVC) and descending aortic (DAo) flow were measured using segmented phase contrast velocity mapping at rest and immediately after exercise to anaerobic threshold on a supine MRI-compatible ergometer. Anaerobic threshold was determined by a prior metabolic exercise test using an upright cycle ergometer. Patients were excluded who could not complete the protocol or had metal artifact preventing these measurements. Systemic blood flow (Qs) was defined as SVC + DAo flow and CollF as Ao - Qs at both resting and exercise conditions. Resting and exercise CollF were indexed to both body surface area and aortic flow and compared using paired student T-test. Indexed CollF was correlated with oxygen consumption (VO2) at peak exercise and anaerobic threshold using linear regression.

## Results

CollF was quantified at both rest and exercise in 31 of 37 pts. CollF was significantly higher at exercise (0.8±0.7 L/min/m^2^) than at rest (0.5±0.4 L/min/m^2^, p=0.003). However, when indexed to aortic flow, there was no significant difference (15±12% at exercise vs. 15±9% at rest). There was no relationship between CollF at rest or exercise and VO2 at AT.

## Conclusions

CollF can be reliably quantified at rest and exercise by comparing aortic flow to systemic flow. CollF increases in proportion to cardiac output at exercise in Fontan patients. There is no apparent relationship between CollF and exercise performance.

## Funding

This research was supported by NIH National Heart, Lung, and Blood Institute grants K23 HL089647 (KKW) and R01 HL67622.

